# Ollivier-Ricci Curvature-Based Method to Community Detection in Complex Networks

**DOI:** 10.1038/s41598-019-46079-x

**Published:** 2019-07-05

**Authors:** Jayson Sia, Edmond Jonckheere, Paul Bogdan

**Affiliations:** Ming Hsieh Department of Electrical Engineering, University of Southern California, Los Angeles, CA 90089 USA

**Keywords:** Computational science, Functional clustering, Electrical and electronic engineering

## Abstract

Identification of community structures in complex network is of crucial importance for understanding the system’s function, organization, robustness and security. Here, we present a novel Ollivier-Ricci curvature (ORC) inspired approach to community identification in complex networks. We demonstrate that the intrinsic geometric underpinning of the ORC offers a natural approach to discover inherent community structures within a network based on interaction among entities. We develop an ORC-based community identification algorithm based on the idea of sequential removal of negatively curved edges symptomatic of high interactions (e.g., traffic, attraction). To illustrate and compare the performance with other community identification methods, we examine the ORC-based algorithm with stochastic block model artificial networks and real-world examples ranging from social to drug-drug interaction networks. The ORC-based algorithm is able to identify communities with either better or comparable performance accuracy and to discover finer hierarchical structures of the network. This opens new geometric avenues for analysis of complex networks dynamics.

## Introduction

Community structures are inherently found in diverse complex networks from technological, biological to social networks. As such, identifying these communities can reveal valuable information regarding the network’s function, structure and organization, and vulnerability. Depending on the type of the network, these communities can represent anything from related web pages in the Internet^1^, functional and chemical pathways in drug-drug interaction networks^2^, to affiliations in social networks^3,4^, to name a few. The community detection of complex networks is an active area of research; however, some consider this an ill-defined problem with no universally accepted definition of what constitutes a “community” nor clear guidelines in assessing its performance. As such, there have been various proposed algorithms utilizing different concepts from edge betweenness, label propagation, to graph modularity.

In this work, we propose a novel geometric approach in network community identification by using the Ollivier-Ricci curvature^5^ (ORC) concept. The notion of curvature, as in Riemannian geometry, quantifies how geodesic paths converge (ORC > 0) or diverge (ORC < 0). The ORC is a coarse version of this concept, and its application to graphs reveals local topological structure and geometry via optimal transport. The ORC captures the notion of network flows of shortest paths via the Wasserstein’s distance formulation wherein a negatively curved edge is a “bottleneck”, along which traffic is intense in a scheme that minimizes the “cost” of transferring “commodities”, while, positively curved edges contribute to transport of “commodities” along with many other edges. Thus, positively curved edges are “well connected”, since none of them are essential for the proper transport operation; therefore, positively curved edges naturally form a “community”. On the other hand, negatively curved edges could be interpreted as “bridges” between communities and cutting them would isolate the network flow between communities. In this context, a “community” is defined as a robust transport of information within the community. Robust means that if some edges are cut information is still going to flow. As an example, for a social network, one hears news not just from one single source but from many different sources. On the other hand, information transfer across communities is more problematic since it relies on these “highway” links that could fail if these connections were removed. The ORC is also recently being applied as a tool in various research areas such as in wireless networking^6,7^, quantum computation^8,9^ as well as robustness analysis of complex networks^10,11^.

## Results

### Ollivier-ricci curvature as a natural metric to discover hierarchies in graphs

The Ollivier-Ricci curvature (ORC) captures two fundamental properties of the structure of complex networks: First, the ORC associated with each edge of the network encodes its shortest path characteristics^6^. Second, the ORC provides information about the frequency of triangles, characterized by the clustering coefficient, within a neighborhood of two adjacent vertices^12,13^ (for mathematical details on how we estimate the ORC for a weighted graph see Methods and Section 2 in Supplementary Information). Starting from these premises, in this work, we aim to address the following questions: Can the ORC help us discover the underlying hierarchical functional characteristics of a complex network? Can the ORC curvature provide algorithmic hints towards solving the hard problem of community identification?

**Figure 1:**
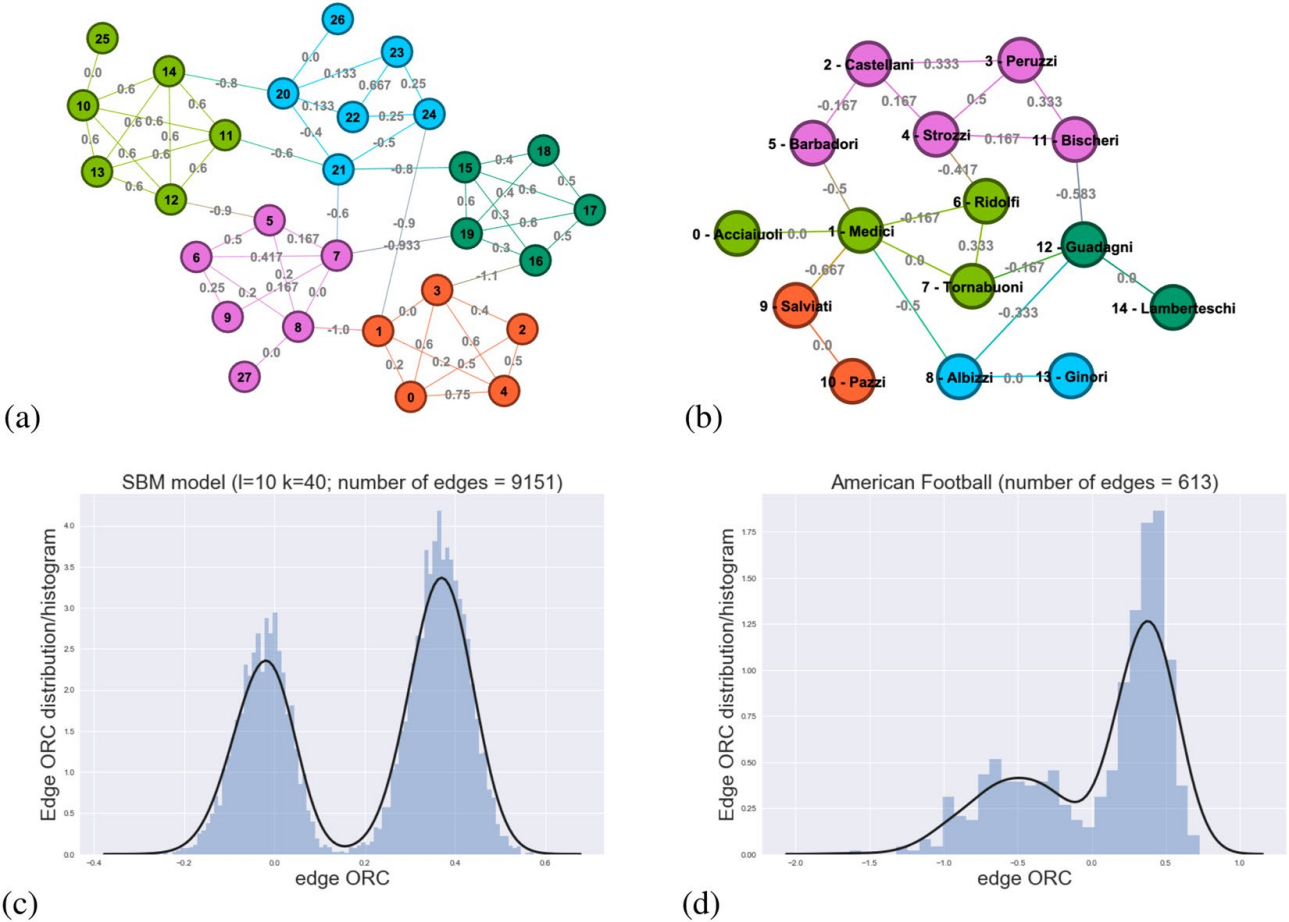
(**a**) An artificial network generated from the stochastic block model (SBM) with the following parameters: size per community *k* = 5, number of communities *l* = 5, intra-cluster probability *p*_*in*_ = 0.8 and inter-cluster probability *p*_*out*_ = 0.05, with added extra leaf nodes (nodes: 25, 26, 27) to illustrate edges with zero curvature, and (**b**) the Florentine family network with edge ORC values shown for both networks. Edge ORC histograms and distribution fit shown for larger networks: (**c**) 800-node SBM artificial network ((*k*, *l*, *p*_*in*_, *p*_*out*_) = (40, 10, 0.7, 0.05)) and (**d**) the American Division IA college football games during regular season of Fall 2000.

To address these questions, we consider an artificial complex network obtained through the stochastic block model (SBM)^14^ as seen in Fig. 1a and a real-world social network (the Florentine family^15^ network) as seen in Fig. 1b. For completeness, Fig. 1c,d illustrate the distribution of edge ORCs for larger networks, one artificial and one real-world network. From Fig. 1a, we make the following observations: (i) edges within a cluster have positive ORC values; (ii) peripheric nodes have zero ORC values; and (iii) edges between clusters have negative ORC values. Range of values for the edge ORC is [−∞, 1]. For networks with clear community structures, especially seen in Fig. 1c,d, the distribution of edge ORC values is clustered into two regions – one region of positive and another region of negative edge ORCs. The high concentration of positive edge ORC values corresponds to the intra-community edges while the concentration of negative edge ORC values corresponds to the inter-community edges. Along the same lines and as can be seen from Fig. 1(a), the positively curved edges form a tight-knit neighborhood of nodes. In contrast, negatively curved edges represent links between tightly connected neighborhoods. These observations suggest that a community identification would proceed by incrementally removing negatively curved edges, and the absence of such negatively curved edges is a natural stopping criterion. We describe the pseudocode and formal analysis of this ORC-based CI algorithm in Methods section and Section 2 of Supplementary Information.

### ORC-based CI detects communities in artificially generated networks

To investigate the accuracy of the proposed ORC-based community identification (CI) algorithm (see Methods Section), we consider a set of artificially constructed complex unweighted networks using the stochastic block model (SBM)^14^. The SBM consists of four parameters, i.e., *k* represents the size of a community, *l* denotes the number of communities, and *p*_*in*_ and *p*_*out*_ are the probability of creating intra- and inter-community edges between any two nodes. Thus, SBM generates a complex network with user-defined community sizes and labels each node accordingly.

**Figure 2:**
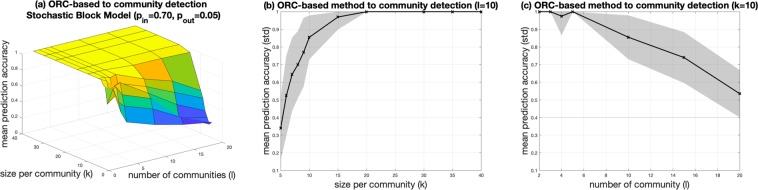
(**a**) Surface plot of the mean prediction accuracy of the ORC-based community detection method for several artificially generated networks using the stochastic block model (SBM) with intra- and inter-community edge wiring probability settings: *p*_*in*_ = 0.70, *p*_*out*_ = 0.05. Prediction mean accuracy with standard deviation bands for (**b**) varying size per community (*k*) parameter for *l* = 10 number of communities, and (**c**) varying number of communities (*l*) parameter for *k* = 10 size per community.

Figure 2a shows the mean prediction accuracy surface plot of the ORC-based CI algorithm applied to artificially generated graphs obtained from the SBM with *p*_*in*_ = 0.7 and *p*_*out*_ = 0.05, respectively. In this study, we vary the number of communities from 2 to 20 and the community size from 5 to 40. From Fig. 2b, we can observe that for a fixed number of communities (*l*), the accuracy improves from 33% for small community sizes to 100% for large community sizes. On the other hand, for fixed size per community, there is a degradation in prediction accuracy as the number of communities is increased. For completeness, Fig. 2b,c show the accuracy and its confidence interval as a function of the community size and the number of communities, respectively.

**Figure 3:**
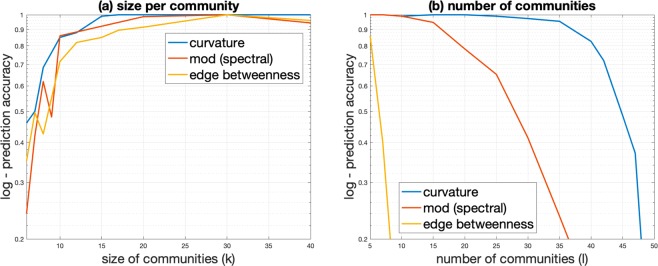
Prediction accuracy of the ORC-based, LEM and EB CIs on SBMs based on varying size per community and number of communities parameters. Intra- and inter-community edge wiring probabilities are set to *p*_*in*_ = 0.7 and *p*_*out*_ = 0.05, respectively. (**a**) Prediction accuracy as a function of size of communities for *l* = 10 number of communities. (**b**) Prediction accuracy as a function of number of communities for *k* = 20 size per community.

We compare the ORC-based CI algorithm with the modularity-based Leading Eigenvalue method^16^ (LEM) and edge betweenness^4^ (EB) based CI algorithms. Figure 3 shows the prediction accuracy for the ORC-, LEM- and EB-based CI algorithms. The accuracy of each algorithm measures the percentage of correctly identified communities from the set of ground truth communities. More precisely, when a set of nodes identified as a community matches all the members of a set from the list of ground truth communities, this is considered a correctly identified community. The prediction accuracy is obtained as a percentage of the correctly identified communities over all ground truth communities. In addition, the results are presented in the context of the SBM detectability regime^17^ which describes the phase transition in the detectability of communities subject to the chosen SBM parameters. For cases when the chosen SBM parameters lie in the undetectable regime, the SBM generated graph is indistinguishable from a random generated graph and community detection is impossible.

As we vary the size per community while keeping the number of communities and the probability of intra- and inter-community edges between any two nodes (*p*_*in*_ and *p*_*out*_) constant (see Fig. 3a), we observe the accuracy of the ORC-based CI improves significantly for networks with more than 7 communities. The low accuracy for sizes *k* less than 7 is due to the high probability to merge two small-sized communities caused by the addition of inter-community edges between the two communities. This observation falls close to the theoretical detectable regime of k^*^ ≥ 3 for varying sizes per community *k* and fixed *l* = 10, *p*_*in*_ = 0.7 and *p*_*out*_ = 0.05. For sizes of community less that 15, the ORC-based CI performs better compared to both LEM and EB methods. There is also a slight degradation in accuracy for the LEM- and EB-based CIs after community size greater than 30.

As we vary the number of communities but keeping the size per community and the probability of intra- and inter-community edges between any two nodes (*p*_*in*_ and *p*_*out*_) constant (see Fig. 3b), we observe the accuracy of the ORC-based CI degrades significantly for networks with more than 35 communities. In contrast, the LEM demonstrates worse performance as its accuracy degrades significantly for more than 15 communities. Both, the ORC- and LEM-based CI reach 50% accuracy for more than 30 communities. The EB-based CI performs the worst out of the three CI algorithms. For varying number of community *l* and fixed *p*_*in*_ = 0.7 and *p*_*out*_ = 0.05, the SBM detectable regime lies in l^*^ ≤ 156.

In short, the ORC-based CI can detect the communities for SBM generated networks even when the communities are not densely connected and even for small community sizes. However, the accuracy degrades when the probability of intra-community edge wiring is less than 0.6 (*p*_*in*_ < 0.6) and the probability of inter-community edge wiring is greater than 0.04 (*p*_*out*_ > 0.04), for fixed size per community and number of communities (*k* = 20 and *l* = 30). In comparison, the accuracy of the LEM-based CI degrades when the probability of intra-community edge wiring is less than 0.7 (*p*_*in*_ = 0.7) and the probability of inter-community edge wiring is greater than 0.03 (*p*_*out*_ > 0.03). For the chosen (*k*, *l*) = (20, 30), the detectability regime lies at ${p}_{in}^{\ast } > 0.35$ for constant *p*_*out*_ = 0.05, and ${p}_{out}^{\ast } < 0.17$ for constant *p*_*in*_ = 0.7. Simulation results show that the ORC-based CI provides better or comparable accuracy with the LEM-based CI (see Section 3 of Supplementary Information for a complete discussion and illustration of results which include the prediction accuracy with respect to the intra- and inter-community wiring probabilities *p*_*in*_ and *p*_*out*_ in the context of the theoretical SBM detectability limit^17^).

### ORC-based method applied to real-world networks

Next, we test the proposed ORC-based CI algorithm to a few real-world network data: Zachary’s Karate Club, American Football games, Political Blogosphere, and DrugBank drug-drug interaction network. We compare the obtained results to other popular established community detection algorithms.

#### Zachary’s karate club

**Figure 4:**
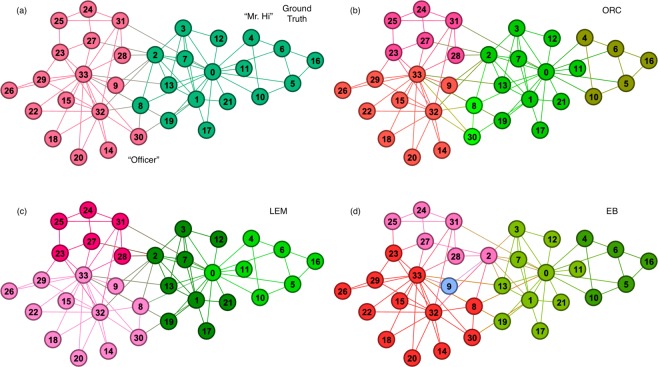
Zachary’s Karate Club. (**a**) Shows the ground truth divided between two communities: ‘Officer’ (in red) and ‘Mr. Hi’ (in green). (**b**–**d**) Show the communities detected (color-coded) using the ORC-, LEM- and EB-based CI methods.

Figure 4 shows the network visualization for the Zachary’s karate club which is a traditional dataset and a standard benchmark in community detection^3^. The network contains 34 nodes (members) and 78 edges (friendships) with an average degree of 4.6. Figure 4a shows the ground truth divided between two communities: ‘Officer’ (in red) and ‘Mr. Hi’ (in green). Figure 4b shows the 5 communities identified by the ORC-based method. Although there are more communities identified, two sub-communities (marked in shades of red) match correctly with the ground-truth subgraph labeled as ‘Officer’ and two sub-communities (marked in shades of green) match correctly with the ground-truth subgraph labeled as ‘Mr. Hi’. The fifth community identified (nodes 8 and 30) neither falls correctly into either communities and thus can be considered as a classification error. From the results, the ORC-based method identifies finer hierarchical structures in the network. More precisely, it identifies further subdivisions within the known truth labeled communities. Figure 4c,d, on the other hand, show that the LEM- and EB-based CI methods identify 4 and 5 communities, respectively. Both assign node 8 as part of the larger community associated with ‘Officer’ which is a classification error. In addition, the EB-based CI has misclassified node 2, apart from labeling node 9 as a separate single-node community.

#### American college football games 2000

**Figure 5:**
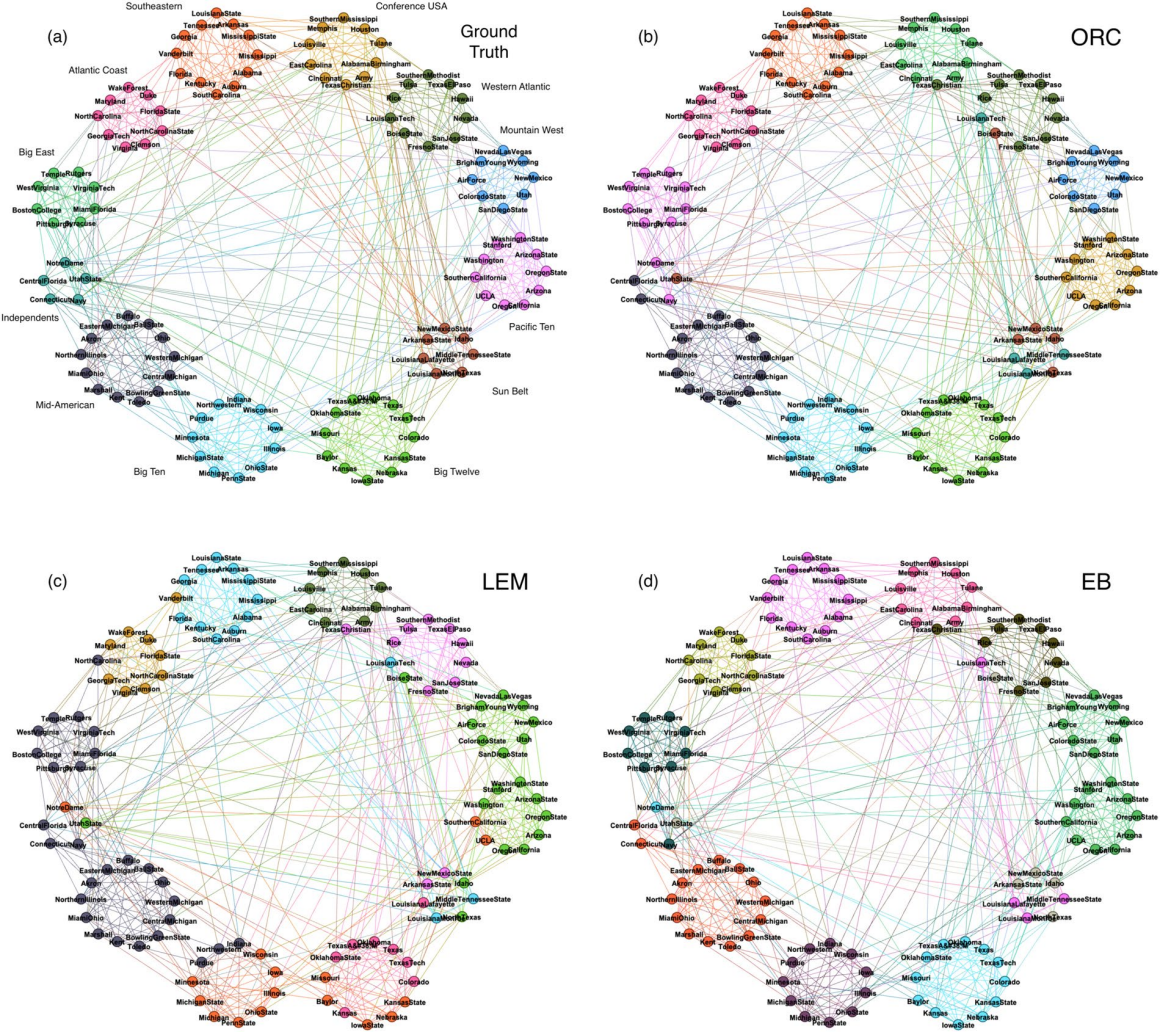
Network of American football games (Division IA) during regular season Fall 2000. (**a**) Each node represents a college color-coded according to its football conference membership (ground truth). (**b**–**d**) Nodes are color-coded according to the communities identified by the (**b**) ORC-, (**c**) LEM-, and (**d**) EB-based CI methods, respectively.

Figure 5a shows the network visualization for the network of American football games between Division IA colleges during regular season of Fall 2000^4^. The colleges are grouped together and color-coded according to their football college conference memberships (e.g. Pac-10, Big-12, etc.). The twelve college football conferences are considered as the ground truth communities. The network contains 115 nodes (colleges) and 613 edges (games) with an average degree of 10. The ORC-based method is able to detect twelve college communities which is also the same number as compared to the list of ground truth football conferences. As seen in Fig. 5b, the ORC-based CI is able to assign all members of 8 out of 12 communities together. If we consider per-node classification accuracy, the ORC-based method incurred 11 misclassified nodes (9.5% misclassification). On the other hand, as seen in Fig. 5c,d, the LEM- and the EB-based CIs are able to identify 8 and 10 college communities, respectively, which are both less than the number of ground truth communities. The per-node classification accuracies are 41 and 19 misclassified nodes translating to 35.7% and 16.5% misclassifications, respectively. In addition, the EB-based CI has identified both ground truth communities “Mountain West” and “Pacific Ten” as one community while the LEM-based CI, in addition to this, has also merged both ground truth communities “Big East” and “Mid-American” as one. Thus, among the three CI methods used, the ORC-based CI performed the best in terms of both correct identification of ground truth communities and per-node community assignments.

Interesting to note that ground truth communities labelled as ‘Independents’ (Notre Dame, Utah State, etc.) and ‘Sun Belt’ (Arkansas State, Idaho, etc.) have no clear community assignments according to three CI methods used. Closer inspection shows that there are very low intra-community links among the members, thus it can be argued that the “Independents” and “Sun Belt” are not really a community in the strict-sense. As another example, Texas Christian is misclassified to “Western Athletic” by all three CI methods but the ground truth assignment is “Conference USA”. Visual inspection of the network shows that Texas Christian has more edge connections (games) with the colleges belonging to the “Western Athletic” compared to those belonging to “Conference USA” which explains the misclassification.

#### Political blogosphere 2005

**Figure 6:**
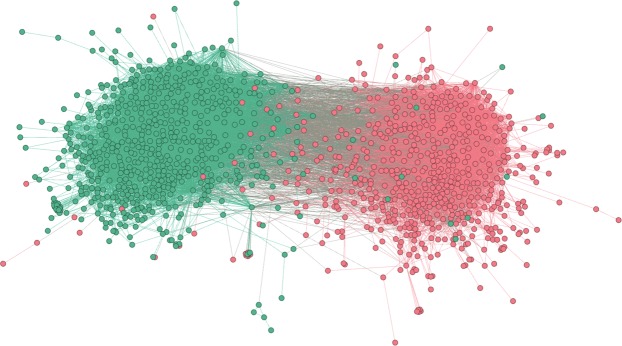
Political Blogosphere 2005 (Nodes = 1222, edges = 16714, average degree = 27.35). Network visualization shows the ground truth communities (green - right/conservative, red - left/liberal).

Figure 6 shows the network visualization for the 2005 Political Blogosphere^1^ which is a network of blog directories labelled according to a blog's political leaning (left/liberal or right/conservative). Considering only the largest connected component, the network contains 1222 nodes (web URLs) and 16714 edges with an average degree of 27.35. Node truth labels indicate political leanings (0 - left/liberal (52.05%); 1 right/conservative (47.95%)). Links between blogs were automatically extracted from a crawl of the front page of the blog. Data on political leaning comes from blog directories with some blogs labeled manually based on the incoming and outgoing links and posts around the time of the 2004 presidential election. The ORC-based method is able to find 146 communities. The top two largest identified communities have sizes 448 and 400 while the rest of the 144 communities have sizes less than 1% of the network size (size 10). Since we know that there are only two ground truth communities, we did preferential attachment of the smaller-sized communities to either of the two largest components based on the inter-community ORC. We identified communities with size 662 (54.17%) for left/liberal and 560 (45.83%) for the right/conservative after preferential attachment of the smaller-sized communities. On the other hand, the LEM-based CI is able to identify two communities with community sizes 677 (55.4%) for left/liberal and 545 (44.6%) for right/conservative. For this dataset, the ORC-based CI performed better than the LEM-based CI in identifying the communities based on the binary ground truth labels.

#### Drug-drug interaction

**Figure 7:**
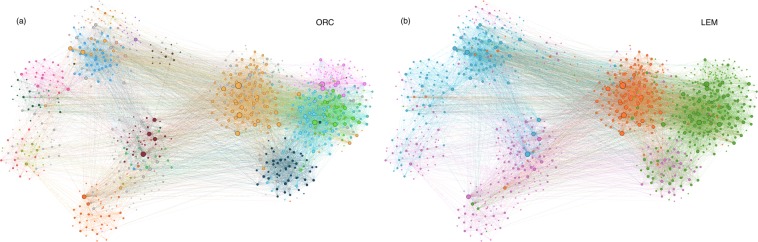
Drug-drug interaction dataset obtained from the DrugBank v4.1 database. Node layout is based on generated topological clusters using Gephi^31^ software with energy-model layout algorithm Force Atlas 2. Nodes are color-coded according to the communities identified by the (**a**) ORC- and (**b**) LEM-based CI methods, respectively.

We also analyze the community structure of drug-drug interaction networks obtained from DrugBank 4.1 database^18^ which was demonstrated as an efficient method for drug repositioning^2^. The network visualization is shown in Fig. 7. Considering only the largest connected component from the dataset, the network contains 1162 nodes (drugs) and 11685 edges (drug interactions) with an average degree of 20. Figure 7a,b show the communities identified by the ORC- and LEM-based CIs, respectively. The ORC-based CI initially identifies 120 communities of which 101 of them have sizes smaller than 1% of the network size. Setting this 1% threshold size limit, we can apply preferential attachment based on the inter-community ORC to merge back these small-scale communities to the larger identified communities. The 19 communities identified by the ORC-based CI are color-coded as shown in Fig. 7a. On the other hand, the LEM-based CI is able to identify 4 communities as shown in Fig. 7b. Comparing the results from Udrescu’s paper^2^, the ORC-based CI matches closely the topological clusters generated based on the energy-model layout algorithm Force Atlas 2, albeit the ORC-based CI identifying 19 communities compared to the 9 labeled topological clusters. Visual inspection of Fig. 7a shows that the ORC-based CI identifies sub-clusters within the topological clusters.

## Discussion

The study of community identification in complex networks is an important and challenging open area of research. Many diverse systems can be represented into networks many of which have inherent community structures. The network abstraction offers a simpler way of looking at a system’s individual elements as nodes and their interactions as edges. In addition, the inherent community structures within networks convey information regarding the system’s function, hierarchy and organization. In the past decade, there have been numerous algorithms^4,16^ proposed to solve the problem of community identification each having its own advantages and limitations. Our proposed ORC-based approach offers a new way to tackle the community identification problem by utilizing the geometric concept of curvature applied to discrete graphs.

From simulations, the ORC-based CI is able to identify communities from the SBM-generated artificial networks with either better or comparable performance accuracies as compared to the LEM- and EB-based CIs. In addition, we also observe that the ORC-based CI performs well in identifying community structures for diverse real-world networks ranging from social to drug-drug interaction networks. For example, the ORC-based CI results for the drug-drug interaction network matches closely the identified topological clustering as compared to the LEM-based modularity method. As seen from the American football and the drug-drug interaction network examples, the LEM-based CI tends to underestimate the total number of communities. One limitation of modularity-based methods is that it has a resolution limit that may prevent it from detecting clusters that are comparatively small compared to the graph as a whole^19^. Contrary to this, the ORC-based CI tends to see the finer subdivisions in the network structures (i.e. identifying “communities within communities”) based on the local topology as quantified by the edge Ollivier-Ricci curvature. Thus, the ORC-based CI tends to identify more communities compared to the number of ground truth communities.

Since the proposed ORC-based CI stems from the idea of successive removal of negatively curved edges, the ORC-based CI will not perform well for networks that have an almost tree-like topology (i.e. graphs that have very few cycles/triangles). This is because tree-like networks have negatively curved edges forming a majority. As a consequence, the ORC-based CI will divide the network into several small communities, thus highly overestimating the number of communities. There are a couple of ways to see this limitation: First, one can argue that tree-like community structures are not considered as a community in the strict-sense since the community members have very few connections among one other. Thus, labelling such communities is considered an ill-defined problem. Second, if the number of communities is known in the first place, preferential attachment heuristics can be applied to re-attach these small-scale communities back to form larger communities.

In conclusion, the ORC-based CI offers a novel alternative solution to the problem of network community identification. Since the algorithm utilizes the geometric concept of network curvature, the ORC-based CI performs particularly well for networks with internally densely-connected community structures. For community structures that are sparsely connected, the ORC-based CI will tend to overestimate the number of communities as it identifies the finer community structure. Preferential attachment heuristics can be applied to merge back these small-scale communities to the larger communities. This preferential attachment portion of the algorithm can be further explored as a future work especially for CI problems with both known and unknown number of communities.

## Methods

### ORC-based community detection algorithm

**Algorithm 1:**
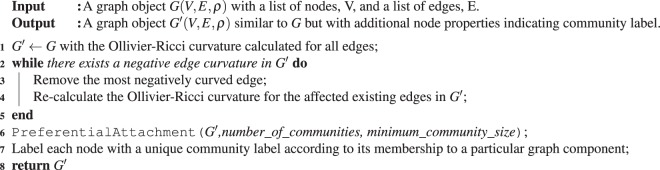
Ollivier-Ricci Curvature based method for Community Detection

We propose the following community detection algorithm which utilizes the concept of Ollivier-Ricci curvature on graphs. The algorithm can be divided into the following steps: (1) calculate the ORC for all edges in the network, (2) remove the most negative ORC edge, (3) re-calculate the edge ORC only for those affected nodes/edges due to prior edge removals, (4) check if all edge curvatures are non-negative, otherwise repeats steps 2 and 3 until condition is satisfied, and (5) perform preferential attachment of isolated graph components if either the number of communities or the minimum accepted community size is known. The pseudo-code of the algorithm is seen in Algorithm 1.

#### Complexity

The time complexity of the proposed ORC-based community detection algorithm boils down to the calculation of the edge ORC of the network. The time complexity to compute the ORC for each edge is essentially the Wasserstein distance computation complexity based on linear programming. Practical run time complexity using network (transportation) simplex algorithm^20^ was shown to be super-cubic. Interior-point or Orlin’s algorithms have complexity of *O*(*V*^3^ log *V*), with *V* as the total number of vertices in the Wasserstein distance sub-problem^21,22^ (Note that *V* depends on twice the average degree of the network typically with $V\ll N$ and $V\ll E$). In the worst case, cycling through each network edge and re-calculating all existing affected edges lead to *O*((*EV*) · *V*^3^ log *V*)). Strategies can be utilized to improved the computation complexity of the proposed algorithm either via a wavelet EMD approximation^21^ of the Wasserstein distance or an ORC bounds analysis^12,23^. The Wasserstein distance computation can be improved from *O*(*V*^3^ log *V*) to *O*(*V*) via the wavelet EMD approximation leading to an overall time complexity of *O*(*EV*^2^) for the proposed algorithm. More information regarding the time complexity of the proposed ORC-based CI and its code implementation are provided in Section 2 of the Supplementary Information.

### Foundation of differential geometry

Fundamental in the process of extending geometry in the Euclidean plane to geometry on a surface ${\mathscr{S}}\subset {{\mathbb{R}}}^{3}$ is the intuitive idea of projecting the ordinary derivative $\frac{d}{dt}X(c(t))$ of a tangent vector field *X*, defined along a curve *c*, on the tangent space to the surface, leading to the concept of Levi-Civita connection

$${\nabla }_{\dot{c}}X\,:={P}_{{T}_{c(t)}{\mathscr{S}}}(\frac{d}{dt}X(c(t)))\in {T}_{c(t)}S.$$

The covariant derivative ∇_*C*_*X* of the vector field *X* along the vector field *C* (not necessarily the tangent to a curve) in a Riemannian manifold ${\mathcal M}$ is a formalization of the intuitive geometric concept of restricting the differential to the tangent space, subject to the additional conditions of symmetry, ∇_*C*_*X* = ∇_*X*_*C*, linearity relative to *C*, the product rule relative to scalar multiplication of *X* and compatibility with the Riemannian metric, viz., $\frac{d}{dt}\langle X(c(t)),Y(c(t))\rangle =\langle {\nabla }_{\dot{c}}X,Y\rangle +\langle X,{\nabla }_{\dot{c}}Y\rangle$.

A vector field *X* is said to be *parallel to itself* along the curve $c:[0,1]\to  {\mathcal M}$, if it satisfies the partial differential equation ${\nabla }_{\dot{c}}X=0$. Under such conditions, *X*(*c*(1)) is said to be a *parallel displacement* of *X*(*c*(0)). This formal definition calls into question by how much this parallel displacement differs from the ordinary Euclidean one. A nonvanishing curvature is precisely symptomatic of such discrepancy. But the immediate problem is that *X*(*c*(0)) and *X*(*c*(1)) lives in different tangent spaces and are difficult to compare. One way to go around this difficulty—challenged by the Ollivier^5^ concept of curvature—is to bring *X*(*c*(1)) back to ${T}_{c(0)} {\mathcal M}$ by another parallel displacement along an extension of *c* to a closed curve. To somewhat simplify the problem without sacrificing generality in our Ollivier-Ricci curvature objective, assume the curve *c* and the vector field *X* live in a 2-dimensional tangent bundle span{*X*, *Y*}. Then

1 $$\angle (X(c(1)),X(c(0))=\,{\rm{Area}}\,(c)K(X,Y),$$

where *K*(*X*, *Y*) is the sectional curvature, a curvature where the parallel displacement is restricted to a 2-dimensional facet. Precisely,

$$K(X,Y)=\frac{\langle R(X,Y)X,Y\rangle }{{\Vert X\Vert }^{2}{\Vert Y\Vert }^{2}-{\langle X,Y\rangle }^{2}},$$

where

$$R(X,Y)={\nabla }_{Y}{\nabla }_{X}-{\nabla }_{X}{\nabla }_{Y}+{\nabla }_{[X,Y]}$$

is the fundamental curvature operator.

#### Connection with wireline networks and diffusion processes

Wireline networks in general send packets along optimal paths, along *geodesics* in Riemannian language. Note that a geodesic is only locally length $\ell (\gamma )={\int }_{\gamma }\,ds$ optimal, as formally the geodesic is defined such that its tangent is parallel to itself, ${\nabla }_{\dot{\gamma }}\dot{\gamma }=0$, where the geodesics is parameterized by arc length and $\dot{\gamma }:=\frac{d\gamma (s)}{ds}$. Motivated by network outages where optimal paths have to be quickly recomputed, the nominal geodesic *γ* is embedded in a family of geodesics, *γ*_*p*_, *p* ∈ (−*ε*, +*ε*) with *γ*_0_ = *γ*. The *Jacobi field* $J(s):={\frac{d}{dp}{\gamma }_{p}(s)|}_{p=0}$, quantifying the variation of geodesics, satisfies the equation

2 $${\nabla }_{\dot{\gamma }}{\nabla }_{\dot{\gamma }}J+K(J,\dot{\gamma })J=0.$$

Under uniform curvature *K*, it is convenient to search a solution of the form *J*(*s*) = *j*(*s*)*W*(*s*), where *W*(*s*) is orthogonal to *γ*(*s*), in which case

3 $$\frac{{d}^{2}}{d{s}^{2}}j(s)+Kj(s)=0.$$

Clearly, if *K* < 0, geodesics are diverging, an observation that lies at the foundation of congestion in wireline Gromov hyperbolic networks^24^.

Other processes of the diffusion type, that is, such processes as heat diffusion and Heat Diffusion wireless networking^6,7,25–28^ involving the Laplace operator, do not “diffuse” along geodesics, but rather follow some thermodynamical-like processes, where the heat kernel exposes the curvature in its Ricci format. The *Ricci curvature* Ric(*X*) is the average of *K*(*X*, *Y*) over all facets span{*X*, *Y*} containing *X*.

Note the fundamental difference between wireline-like networking and diffusion. Wireline networking involves large-scale optimal paths, whereas wireless networking in both its backpressure and Heat Diffusion implementations is driven by strictly local queue backlogs, in the same way as heat diffusion is driven by a strictly local temperature gradient.

#### Towards ollivier-ricci curvature

Contrary to what is usually done, here, we attempt to define curvature by reference to different tangent spaces, one centered at *γ*(0), the other at *γ*(*ε*). Consider two *δ*-radius balls *B*_*γ*(0)_, *B*_*γ*(*ε*)_. We establish a correspondence between the two balls as follows: Consider *x* ∈ *B*_*γ*(0)_ along with $X={\exp }_{\gamma (0)}^{-1}(x)$. Displace *X* parallel to itself along *γ* from *γ*(0) to *γ*(*ε*) to obtain *Y*. Define *y* = exp_*γ*(*ε*)_(*Y*). This establishes the correspondence $T:x\mapsto y$. To introduce a *transport* idea, the ball *B*_*γ*(0)_ is endowed with a probability measure *μ*_0_ and *dμ*_0_(*x*) is transported to *y* = *T*(*x*) along a geodesic arc [*x*, *y*] of length equal to the distance *d*(*x*, *y*).

Invoking the Jacobi field (2 and 3), the distance *d*(*x*, *T*(*x*)) along the “perturbed” geodesic [*x*, *y*] and how it relates to the distance *d*(*γ*(0), *γ*(ε) = *ε*) along the “nominal” geodesic depends on the sectional curvature $K(X,\dot{\gamma })$. Therefore, the cost of the transport

4 $$C(T)={\int }_{{B}_{\gamma (0)}}\,d(x,T(x))d{\mu }_{0}(x),$$

since it involves an integral over all *x* ∈ *B*_*γ*(0)_, tacitly involves an integral over all tangent vectors *X* ∈ *T*_*γ*(0)_*B*_*γ*(0)_ and as such averages $K(X,\dot{\gamma })$ over all *X* to yield the Ricci curvature ${\rm{Ric}}{}_{\dot{\gamma }(0)}( {\mathcal M} )$.

In 0-curvature, the distance *d*(*x*, *T*(*x*)) is independent of *x* and therefore the transport cost is *d*(*γ*(0), *γ*(*ε*) = *ε*). It remains to see how this distance is affected by the curvature. Define *dθ*(*s*) to be the elementary angle swept by the normal *W*(*s*) to the geodesic under an elementary move *ds* along such geodesic. Then

$$d(x,T(x))=\varepsilon +{\int }_{0}^{\varepsilon }j(s)d\theta (s).$$

*j*(*s*) is the distance between the nominal and perturbed geodesics measured along the normal to the nominal geodesic; using (3), it is evaluated as

$$\begin{array}{rcl}j(s) & = & \delta \,\cosh \,(\sqrt{-K}s)-\frac{\varepsilon \delta }{2}\sqrt{-K}\,\sinh \,(\sqrt{-K}s)\\  & \approx  & \delta \,\cosh \,(\sqrt{-K}s).\end{array}$$

Next, we apply (1) to the closed path made up with ${\dot{\gamma }}_{0}(s)ds$, *j*(*s* + *ds*)*W*(*s* + *ds*), $-\,{\dot{\gamma }}_{\delta }(s+ds)ds$ and −*j*(*s*)*W*(*s*). Noting that the left-hand side of (1) is the full discrepancy angle around the closed path while we only need the discrepancy along the nominal geodesic, we get

5 $$d\theta =\frac{1}{2}d\,{\rm{Area}}\,(j,ds)\sqrt{-K}$$

6 $$=\,\frac{1}{2}j(s)ds\sqrt{-K}.$$

Putting everything together and after an elementary integration, it is found that

$$d(x,T(x))\approx \varepsilon (1-\frac{1}{2}K{\delta }^{2}),$$

an estimate consistent with that of [5, Prop. 6, Sec. 8].

The above estimate was derived nominally in a negatively curved manifold, but redeveloping the same argument with ordinary trigonometry rather than hyperbolic trigonometry would validate it in positively curved spaces.

The above clearly indicates that in negative curvature, the transportation cost from *x* to *T*(*x*) is larger than along the nominal geodesic. In positive curvature, the *x* to *T*(*x*) cost is smaller than along *γ*.

To summarize:

$$\begin{array}{rcl}{{\rm{Ric}}}_{\dot{\gamma }(0)}( {\mathcal M} ) < 0 & \iff  & {\int }_{{B}_{\gamma (0)}}d(x,T(x))d{\mu }_{0}(x) > d(\gamma (0),\gamma (\varepsilon )),\\ {{\rm{Ric}}}_{\dot{\gamma }(0)}( {\mathcal M} )=0 & \iff  & {\int }_{{B}_{\gamma (0)}}d(x,T(x))d{\mu }_{0}(x)=d(\gamma (0),\gamma (\varepsilon )),\\ {{\rm{Ric}}}_{\dot{\gamma }(0)}( {\mathcal M} ) > 0 & \iff  & {\int }_{{B}_{\gamma (0)}}d(x,T(x))d{\mu }_{0}(x) < d(\gamma (0),\gamma (\varepsilon )).\end{array}$$

#### From riemannian manifolds to graphs

On a graph ${\mathscr{G}}=({\mathscr{V}}, {\mathcal E} )$ endowed with a distance $d(\cdot \,,\,\cdot )$, we need to emulate the Riemannian manifold environment. We identify an edge *ij* of the graph with the geodesic *γ*([0*, ε*]) and the graph theoretic neighborhoods ${{\mathscr{N}}}_{i}$, ${{\mathscr{N}}}_{j}$ of *i* and *j* with the balls *B*_*γ*(0*)*,_ *B*_*γ*(*ε*)_ centered at *γ(*0)*, γ*(*ε*). Discrete probabilit_*i*_es *μ*_*i*_, *μ*_*j*_ on ${{\mathscr{N}}}_{i}$, ${{\mathscr{N}}}_{j}$ are obvious substitutes for the measures *μ*_0_, *μ*_*ε*_ on the balls *B*_*γ(*0*)*,_ *B*_*γ*(*ε*)_.

The difficulty is to emulate the Riemannian connection resorting only to the graph theoretic distance, or at the very least redefine the cost *C*(*T*) in (4) in a way that does not involve parallel displacement. Proceeding from

$$C=\mathop{{\rm{\inf }}}\limits_{T:{B}_{\gamma (0)}\to {B}_{\gamma (\varepsilon )}}{\int }_{{B}_{\gamma (0)}}d(x,T(x))d{\mu }_{0}(x),$$

where *T* is restricted to be one-to-one, the graph theoretic emulation of the above is

$${\overrightarrow{C}}_{{\mathscr{G}}}=\mathop{{\rm{\min }}}\limits_{{{\mathscr{N}}}_{i}\ni k\mapsto \ell \in {{\mathscr{N}}}_{j}}\sum _{k\in {{\mathscr{N}}}_{i}}\,d(k,\ell ){\mu }_{i}(k)$$

In this case, because the cardinalities of ${{\mathscr{N}}}_{i}$ and ${{\mathscr{N}}}_{j}$ might not be the same, the mapping $k\mapsto \ell$, while one-to-many, could be many-to-one. As such, the formula lacks symmetry and cannot be used as a Wasserstein-like distance. To remedy this situation, we introduce a *transference plan* ${\xi }^{ij}(k,\ell )$ as a substitute for the many-to-many mapping $k\mapsto \ell$, with the added generality that only a piece ${\xi }^{ij}(k,\ell )$ of *μ*_*i*_(*k*) is transferred to $\ell$. The above formula hence becomes

$${C}_{{\mathscr{G}}}=\mathop{{\rm{\min }}}\limits_{{\xi }^{ij}(k,\ell )}\sum _{k\in {{\mathscr{N}}}_{i},\ell \in {{\mathscr{N}}}_{j}}\,d(k,\ell ){\xi }^{ij}(k,\ell )$$

with of course the consistency conditions

$$\sum _{\ell \in {{\mathscr{N}}}_{j}}\,{\xi }^{ij}(k,\ell )={\mu }_{i}(k),\,\sum _{k\in \in {{\mathscr{N}}}_{i}}\,{\xi }^{ij}(k,\ell )={\mu }_{j}(\ell ).$$

The curvature concept that emanates from this cost (${C}_{{\mathscr{G}}} > ( < )\varepsilon \iff {\rm{Ric}} < ( > )0$) is very local, around an edge, in contradiction with the global Gromov concept. This explains why such concept appears the correct one to anticipate performance of backpressure and Heat Diffusion protocols on wireless networks^6,7^.

### Ollivier-ricci curvature on complex networks

The proposed community detection algorithm utilizes the coarse Ricci curvature, referred to as Ollivier-Ricci curvature, in its version designed for complex networks. Since the Ricci curvature involves a privileged direction ($\dot{\gamma }(0)$ on ${\mathcal M}$, edge *ij* on ${\mathscr{G}}$), it incorporates a generic concept of *flow*. In the Riemannian model, ${{\rm{Ric}}}_{\dot{\gamma }(0)} < 0$ means “heavy” flow, in the sense that the least cost transport of probability mass takes the geodesic *γ* path rather than being distributed along the perturbed geodesics. In the graph/network context, the ball of mass around *i* is the set of neighbors of *i* (same for *j*). Similarly, the idea is to find the best way to transfer the ball of mass around the vertex *i* to that around the vertex *j*.

Consider a weighted graph $(({\mathscr{V}}, {\mathcal E} ),\rho )$. On this graph, over each vertex *i*, we define a probability measure on ${{\mathscr{N}}}_{i}:=\{k\in {\mathscr{V}}:ik\in  {\mathcal E} \}$ as follows:

$$\begin{array}{rcl}{\mu }_{i}(k) & = & \frac{{\rho }_{ik}}{\sum _{k\in {\mathscr{N}}(i)}\,{\rho }_{ik}},\,\,{\rm{if}}\,ik\in  {\mathcal E} \\  & = & 0\,{\rm{otherwise}}\end{array}$$

The Ollivier-Ricci curvature with the set of probability measures $\{{\mu }_{i}:i\in {\mathscr{V}}\}$ is defined along the geodesic path [*i*, *j*] as

7 $$\kappa ([i,j])=1-\frac{{W}_{1}({\mu }_{i},{\mu }_{j})}{d(i,j)},$$

where *W*_1_(*μ*_*i*_, *μ*_*j*_) is the first Wasserstein distance between the probability measures *μ*_*i*_ and *μ*_*j*_ defined on ${{\mathscr{N}}}_{i}$ and ${{\mathscr{N}}}_{j}$, respectively, and is defined as

8 $${W}_{1}({\mu }_{i},{\mu }_{j})={\rm{\inf }}\sum _{k,\ell \in {{\mathscr{N}}}_{i}\times {{\mathscr{N}}}_{j}}\,d(k,\ell ){\xi }^{ij}(k,\ell ).$$

The infimum is extended over all “coupling” measure ${\xi }^{ij}(k,\ell )$ defined on ${{\mathscr{N}}}_{i}\times {{\mathscr{N}}}_{j}$ and projecting on the first(second) factor as *μ*_*i*_(*μ*_*j*_). More intuitively, ${\xi }^{ij}(k,\ell )$ is called *transference plan*. It tells us how much of the mass of *k* is transferred to $\ell$, but it does not tell us anything about the actual path that the mass has to follow. $d(i,j)$ is the usual (distance) metric emanating from the edge weight *ρ*. The first Wasserstein distance *W*_1_ is also referred to as the Earth Mover’s Distance (EMD) in computer science applications.

Exact computation of the Ollivier-Ricci curvature can be computed via calculation of the Wasserstein distance using linear programming^6^ and parallel computation^29,30^.

## Supplementary information

Supplementary Information
