# Supplementary material for: Ollivier-Ricci Curvature-Based Method to Community Detection in Complex Networks
Source: Sci Rep. 2019 Jul 5;9:9800. doi: 10.1038/s41598-019-46079-x (PMC6611887; doi:10.1038/s41598-019-46079-x)
Supplement: Supplementary file 1 — Supplementary Information [file 41598_2019_46079_MOESM1_ESM.pdf]

Supplementary Information to Ollivier-Ricci Curvature-Based Method to Community Detection in Complex Networks

Jayson Sia, Edmond Jonckheere and Paul Bogdan

May 23, 2019

Contents

1 Coarse Geometry of Complex Networks **1**
- 1.1 Ollivier-Ricci curvature . . . 1
  - 1.1.1 Foundation of differential geometry . . . 1
  - 1.1.2 Connection with wireline networks and diffusion processes . . . 2
  - 1.1.3 Towards Ollivier-Ricci curvature . . . 2
  - 1.1.4 From Riemannian manifolds to graphs . . . 3
- 1.2 Ollivier-Ricci curvature of graphs . . . 4

2 Proposed Algorithm **5**
- 2.1 Ollivier-Ricci curvature based community detection algorithm . . . 5
- 2.2 Code implementation . . . 5
- 2.3 Computational time complexity analysis . . . 6
  - 2.3.1 Ollivier-Ricci curvature wavelet EMD approximation . . . 6
  - 2.3.2 Ollivier-Ricci curvature bounds . . . 6
  - 2.3.3 Time complexity table . . . 7

3 Additional Results and Discussion **7**
- 3.1 Stochastic Block Model (SBM) . . . 7
  - 3.1.1 Intra- and inter-cluster densities vs. prediction accuracy . . . 7
  - 3.1.2 Detectability regime in stochastic block models . . . 9

1 Coarse Geometry of Complex Networks

1.1 Ollivier-Ricci curvature

1.1.1 Foundation of differential geometry

Fundamental in the process of extending geometry in the Euclidean plane to geometry on a surface $\mathcal{S} \subset \mathbb{R}^3$ is the intuitive idea of projecting the ordinary derivative $\frac{d}{dt}X(c(t))$ of a tangent vector field X, defined along a curve c, on the tangent space to the surface, leading to the concept of Levi-Civita connection

$$\nabla_{\dot{c}}X := P_{T_{c(t)}\mathcal{S}}\left(\frac{d}{dt}X(c(t))\right) \in T_{c(t)}S.$$

The covariant derivative $\nabla_C X$ of the vector field X along the vector field C (not necessarily the tangent to a curve) in a Riemannian manifold $\mathcal{M}$ is a formalization of the intuitive geometric concept of restricting the differential to the tangent space, subject to the additional conditions of symmetry, $\nabla_C X = \nabla_X C$, linearity relative to C, the product rule relative to scalar multiplication of X and compatibility with the Riemannian metric, viz., $\frac{d}{dt}\langle X(c(t)), Y(c(t))\rangle = \langle \nabla_{\dot{c}}X, Y\rangle + \langle X, \nabla_{\dot{c}}Y\rangle$.

A vector field X is said to be *parallel to itself* along the curve $c : [0,1] \to \mathcal{M}$, if it satisfies the partial differential equation $\nabla_{\dot{c}}X = 0$. Under such conditions, $X(c(1))$ is said to be a

parallel displacement of $X(c(0))$. This formal definition calls into question by how much this parallel displacement differs from the ordinary Euclidean one. A nonvanishing curvature is precisely symptomatic of such discrepancy. But the immediate problem is that $X(c(0))$ and $X(c(1))$ lives in different tangent spaces and are difficult to compare. One way to go around this difficulty—challenged by the Ollivier [Oll09] concept of curvature—is to bring $X(c(1))$ back to $T_{c(0)}\mathcal{M}$ by another parallel displacement along an extension of c to a closed curve. To somewhat simplify the problem without sacrificing generality in our Ollivier-Ricci curvature objective, assume the curve c and the vector field X live in a 2-dimensional tangent bundle span$\{X,Y\}$. Then

$$\angle(X(c(1)), X(c(0)) = \text{Area}(c)K(X,Y), \tag{1}$$

where $K(X,Y)$ is the sectional curvature, a curvature where the parallel displacement is restricted to a 2-dimensional facet. Precisely,

$$K(X,Y) = \frac{\langle R(X,Y)X, Y\rangle}{\|X\|^2\|Y\|^2 - \langle X,Y\rangle^2},$$

where

$$R(X,Y) = \nabla_Y\nabla_X - \nabla_X\nabla_Y + \nabla_{[X,Y]}$$

is the fundamental curvature operator.

1.1.2 Connection with wireline networks and diffusion processes

Wireline networks in general send packets along optimal paths, along *geodesics* in Riemannian language. Note that a geodesic is only locally length $\ell(\gamma) = \int_\gamma ds$ optimal, as formally the geodesic is defined such that its tangent is parallel to itself, $\nabla_{\dot\gamma}\dot\gamma = 0$, where the geodesics is parameterized by arc length and $\dot\gamma := \frac{d\gamma(s)}{ds}$. Motivated by network outages where optimal paths have to be quickly recomputed, the nominal geodesic γ is embedded in a family of geodesics, γ_p, $p \in (-\epsilon, +\epsilon)$ with $\gamma_0 = \gamma$. The *Jacobi field* $J(s) := \frac{d}{dp}\gamma_p(s)\Big|_{p=0}$, quantifying the variation of geodesics, satisfies the equation

$$\nabla_{\dot\gamma}\nabla_{\dot\gamma}J + K(J,\dot\gamma)J = 0. \tag{2}$$

Under uniform curvature K, it is convenient to search a solution of the form $J(s) = j(s)W(s)$, where $W(s)$ is orthogonal to $\gamma(s)$, in which case

$$\frac{d^2}{ds^2}j(s) + Kj(s) = 0. \tag{3}$$

Clearly, if $K < 0$, geodesics are diverging, an observation that lies at the foundation of congestion in wireline Gromov hyperbolic networks [JLBB11].

Other processes of the diffusion type, that is, such processes as heat diffusion and Heat Diffusion wireless networking [BJK12, BJK14a, BJK14b, BJK14c, WJB14, WJB16] involving the Laplace operator, do not "diffuse" along geodesics, but rather follow some thermodynamical-like processes, where the heat kernel exposes the curvature in its Ricci format. The *Ricci curvature* Ric(X) is the average of $K(X,Y)$ over all facets span$\{X,Y\}$ containing X.

Note the fundamental difference between wireline-like networking and diffusion. Wireline networking involves large-scale optimal paths, whereas wireless networking in both its backpressure and Heat Diffusion implementations is driven by strictly local queue backlogs, in the same way as heat diffusion is driven by a strictly local temperature gradient.

1.1.3 Towards Ollivier-Ricci curvature

Contrary to what is usually done, here, we attempt to define curvature by reference to different tangent spaces, one centered at $\gamma(0)$, the other at $\gamma(\epsilon)$. Consider two δ-radius balls $B_{\gamma(0)}$, $B_{\gamma(\epsilon)}$. We establish a correspondence between the two balls as follows: Consider $x \in B_{\gamma(0)}$ along with $X = \exp^{-1}_{\gamma(0)}(x)$. Displace X parallel to itself along γ from $\gamma(0)$ to $\gamma(\epsilon)$ to obtain Y. Define $y = \exp_{\gamma(\epsilon)}(Y)$. This establishes the correspondence $T : x \mapsto y$. To introduce a *transport* idea, the ball $B_{\gamma(0)}$ is endowed with a probability measure μ_0 and $d\mu_0(x)$ is transported to $y = T(x)$ along a geodesic arc $[x,y]$ of length equal to the distance $d(x,y)$.

Invoking the Jacobi field (2)-(3), the distance $d(x, T(x))$ along the "perturbed" geodesic $[x, y]$ and how it relates to the distance $d(\gamma(0), \gamma(\epsilon)) = \epsilon$ along the "nominal" geodesic depends on the sectional curvature $K(X, \dot{\gamma})$. Therefore, the cost of the transport

$$C(T) = \int_{B_{\gamma(0)}} d(x, T(x)) d\mu_0(x), \tag{4}$$

since it involves an integral over all $x \in B_{\gamma(0)}$, tacitly involves an integral over all tangent vectors $X \in T_{\gamma(0)} B_{\gamma(0)}$ and as such averages $K(X, \dot{\gamma})$ over all X to yield the Ricci curvature $\mathrm{Ric}_{\dot{\gamma}(0)}(\mathcal{M})$.

In 0-curvature, the distance $d(x, T(x))$ is independent of x and therefore the transport cost is $d(\gamma(0), \gamma(\epsilon)) = \epsilon$. It remains to see how this distance is affected by the curvature. Define $d\theta(s)$ to be the elementary angle swept by the normal $W(s)$ to the geodesic under an elementary move ds along such geodesic. Then

$$d(x, T(x)) = \epsilon + \int_0^\epsilon j(s) d\theta(s).$$

$j(s)$ is the distance between the nominal and perturbed geodesics measured along the normal to the nominal geodesic; using (3), it is evaluated as

$$\begin{aligned} j(s) &= \delta \cosh(\sqrt{-K} s) - \frac{\epsilon\delta}{2} \sqrt{-K} \sinh(\sqrt{-K} s) \\ &\approx \delta \cosh(\sqrt{-K} s). \end{aligned}$$

Next, we apply (1) to the closed path made up with $\dot{\gamma}_0(s)ds$, $j(s+ds)W(s+ds)$, $-\dot{\gamma_\delta}(s+ds)ds$ and $-j(s)W(s)$. Noting that the left-hand side of (1) is the full discrepancy angle around the closed path while we only need the discrepancy along the nominal geodesic, we get

$$d\theta = \frac{1}{2} d\mathrm{Area}(j, ds) \sqrt{-K} \tag{5}$$

$$= \frac{1}{2} j(s) ds \sqrt{-K}. \tag{6}$$

Putting everything together and after an elementary integration, it is found that

$$d(x, T(x)) \approx \epsilon \left(1 - \frac{1}{2} K \delta^2 \right),$$

an estimate consistent with that of [Oll09, Prop. 6, Sec. 8].

The above estimate was derived nominally in a negatively curved manifold, but redeveloping the same argument with ordinary trigonometry rather than hyperbolic trigonometry would validate it in positively curved spaces.

The above clearly indicates that in negative curvature, the transportation cost from x to $T(x)$ is larger than along the nominal geodesic. In positive curvature, the x to $T(x)$ cost is smaller than along γ.

To summarize:

$$\begin{aligned} \mathrm{Ric}_{\dot{\gamma}(0)}(\mathcal{M}) < 0 &\Leftrightarrow \textstyle\int_{B_{\gamma(0)}} d(x, T(x)) d\mu_0(x) > d(\gamma(0), \gamma(\epsilon)), \\ \mathrm{Ric}_{\dot{\gamma}(0)}(\mathcal{M}) = 0 &\Leftrightarrow \textstyle\int_{B_{\gamma(0)}} d(x, T(x)) d\mu_0(x) = d(\gamma(0), \gamma(\epsilon)), \\ \mathrm{Ric}_{\dot{\gamma}(0)}(\mathcal{M}) > 0 &\Leftrightarrow \textstyle\int_{B_{\gamma(0)}} d(x, T(x)) d\mu_0(x) < d(\gamma(0), \gamma(\epsilon)). \end{aligned}$$

1.1.4 From Riemannian manifolds to graphs

On a graph $\mathcal{G} = (\mathcal{V}, \mathcal{E})$ endowed with a distance $d(\cdot, \cdot)$, we need to emulate the Riemannian manifold environment. We identify an edge ij of the graph with the geodesic $\gamma([0, \epsilon])$ and the graph theoretic neighborhoods $\mathcal{N}_i$, $\mathcal{N}_j$ of i and j with the balls $B_{\gamma(0)}$, $B_{\gamma(\epsilon)}$ centered at $\gamma(0)$, $\gamma(\epsilon)$. Discrete probabilities μ_i, μ_j on $\mathcal{N}_i$, $\mathcal{N}_j$ are obvious substitutes for the measures μ_0, μ_ϵ on the balls $B_{\gamma(0)}$, $B_{\gamma(\epsilon)}$.

The difficulty is to emulate the Riemannian connection resorting only to the graph theoretic distance, or at the very least redefine the cost $C(T)$ in (4) in a way that does not involve parallel displacement. Proceeding from

$$C = \inf_{T: B_{\gamma(0)} \to B_{\gamma(\epsilon)}} \int_{B_{\gamma(0)}} d(x, T(x)) d\mu_0(x),$$

where T is restricted to be one-to-one, the graph theoretic emulation of the above is

$$\vec{C}_{\mathcal{G}} = \min_{\mathcal{N}_i \ni k \mapsto \ell \in \mathcal{N}_j} \sum_{k \in \mathcal{N}_i} d(k,\ell)\mu_i(k)$$

In this case, because the cardinalities of $\mathcal{N}_i$ and $\mathcal{N}_j$ might not be the same, the mapping $k \mapsto \ell$, while one-to-many, could be many-to-one. As such, the formula lacks symmetry and cannot be used as a Wasserstein-like distance. To remedy this situation, we introduce a *transference plan* $\xi^{ij}(k,\ell)$ as a substitute for the many-to-many mapping $k \mapsto \ell$, with the added generality that only a piece $\xi^{ij}(k,\ell)$ of $\mu_i(k)$ is transferred to ℓ. The above formula hence becomes

$$C_{\mathcal{G}} = \min_{\xi^{ij}(k,\ell)} \sum_{k \in \mathcal{N}_i, \ell \in \mathcal{N}_j} d(k,\ell)\xi^{ij}(k,\ell)$$

with of course the consistency conditions

$$\sum_{\ell \in \mathcal{N}_j} \xi^{ij}(k,\ell) = \mu_i(k), \qquad \sum_{k \in \in \mathcal{N}_i} \xi^{ij}(k,\ell) = \mu_j(\ell).$$

The curvature concept that emanates from this cost ($C_{\mathcal{G}} > (<)\epsilon \Leftrightarrow \mathrm{Ric} < (>)0$) is very local, around an edge, in contradiction with the global Gromov concept. This explains why such concept appears the correct one to anticipate performance of backpressure and Heat Diffusion protocols on wireless networks [WJB14, WJB16].

1.2 Ollivier-Ricci curvature of graphs

The proposed community detection algorithm utilizes the coarse Ricci curvature, referred to as Ollivier-Ricci curvature, in its version designed for complex networks. Since the Ricci curvature involves a privileged direction ($\dot{\gamma}(0)$ on $\mathcal{M}$, edge ij on $\mathcal{G}$), it incorporates a generic concept of *flow*. In the Riemannian model, $\mathrm{Ric}_{\dot{\gamma}(0)} < 0$ means "heavy" flow, in the sense that the least cost transport of probability mass takes the geodesic γ path rather than being distributed along the perturbed geodesics. In the graph/network context, the ball of mass around i is the set of neighbors of i (same for j). Similarly, the idea is to find the best way to transfer the ball of mass around the vertex i to that around the vertex j.

Consider a weighted graph $((\mathcal{V},\mathcal{E}),\rho)$. On this graph, over each vertex i, we define a probability measure on $\mathcal{N}_i := \{k \in \mathcal{V} : ik \in \mathcal{E}\}$ as follows:

$$\begin{aligned} \mu_i(k) &= \frac{\rho_{ik}}{\sum_{k \in \mathcal{N}(i)} \rho_{ik}}, \quad \text{if } ik \in \mathcal{E} \\ &= 0 \quad \text{otherwise} \end{aligned}$$

The Ollivier-Ricci curvature with the set of probability measures $\{\mu_i : i \in \mathcal{V}\}$ is defined along the geodesic path $[i,j]$ as

$$\kappa([i,j]) = 1 - \frac{W_1(\mu_i,\mu_j)}{d(i,j)}, \tag{7}$$

where $W_1(\mu_i,\mu_j)$ is the first Wasserstein distance between the probability measures μ_i and μ_j defined on $\mathcal{N}_i$ and $\mathcal{N}_j$, respectively, and is defined as

$$W_1(\mu_i,\mu_j) = \inf \sum_{k,\ell \in \mathcal{N}_i \times \mathcal{N}_j} d(k,\ell)\xi^{ij}(k,\ell). \tag{8}$$

The infimum is extended over all "coupling" measure $\xi^{ij}(k,\ell)$ defined on $\mathcal{N}_i \times \mathcal{N}_j$ and projecting on the first(second) factor as $\mu_i(\mu_j)$. More intuitively, $\xi^{ij}(k,\ell)$ is called *transference plan*. It tells us how much of the mass of k is transferred to ℓ, but it does not tell us anything about the actual path that the mass has to follow. $d(i,j)$ is the usual (distance) metric emanating from the edge weight ρ. The first Wasserstein distance W_1 is also referred to as the Earth Mover's Distance (EMD) in computer science applications.

Exact computation of the Ollivier-Ricci curvature can be computed via calculation of the Wasserstein distance using linear programming[WJB14] and parallel computation[LRO+17, NLG+15]. Fig. 1 shows an illustration of the mass transport from ball of mass concentrated around node x to a ball of mass concentrated at node y.

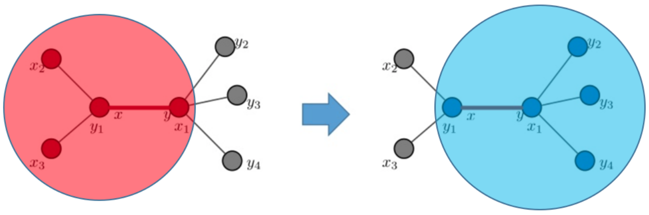

Figure 1: Ollivier-Ricci curvature with ball of mass concentrated around node x and its neighbors (shown in red) that is transferred to the ball of mass concentrated around node y and its neighbors (shown in blue).

2 Proposed Algorithm

2.1 Ollivier-Ricci curvature based community detection algorithm

Algorithm 1: Ollivier-Ricci Curvature based method for Community Detection

Input : A graph object $G(V, E, \rho)$ with a list of nodes, V, and a list of edges, E.
Output : A graph object $G'(V, E, \rho)$ similar to G but with additional node properties indicating community label.

$G' \leftarrow G$ with the Ollivier-Ricci curvature calculated for all edges;
while *there exists a negative edge curvature in* G' **do**
  Remove the most negatively curved edge;
  Re-calculate the Ollivier-Ricci curvature for the affected existing edges in G';
end
`PreferentialAttachment`(G',*number_of_communities, minimum_community_size*);
Label each node with a unique community label according to its membership to a particular graph component;
return G'

We propose the following community detection algorithm which utilizes the concept of Ollivier-Ricci curvature on graphs.

1. Calculate the Ollivier-Ricci curvature for all edges in the Graph
2. Remove the most negatively curved edge
3. Re-calculate the Ollivier-Ricci curvature for the remaining edges in the Graph
   - Re-calculate the curvature only for those affected nodes/edges due to previous edge removal. Not all edge curvatures need to be re-calculated. This significantly reduce running time in between iterations.
4. Check if all edge curvatures are non-negative, otherwise repeats Steps 2 and 3 until the condition is satisfied.
5. perform preferential attachment of isolated graph components if either the number of communities or the minimum accepted community size is known.

The pseudocode for the proposed ORC-based CI is shown in Algorithm 1.

2.2 Code implementation

All community detection algorithms used in the text are implemented in python 3.6. The ORC-based CI is implemented using `GraphRicciCurvature` package[NLG+15] (`https://github.com/saibalmars/GraphRicciCurvature`) for the calculation of the edge Ollivier-Ricci curvature. The multiprocessing option is utilized to speed up the computation time via parallel computation by using as much available CPU cores as possible. The python packages `networkx`[HSS08] and `igraph`[CN14] are used for the generation of the artificial and real-world graphs as well as for the

implementation of the (modularity-based) leading eigenvalue and edge-betweenness community detection methods. The Gephi[BHJ09] software is used for the graph visualization of the chosen real-world networks in the main text.

2.3 Computational time complexity analysis

Regarding the time complexity of the proposed algorithm, the following two subsections describe two strategies for improving it by either a wavelet EMD approximation of the first Wasserstein distance computation and via the ORC bounds analysis.

2.3.1 Ollivier-Ricci curvature wavelet EMD approximation

The proposed Ollivier-Ricci curvature (ORC)-based community identification algorithm relies on the iterative computation of the edge ORC values. Computation of the edge ORC is essentially a linear programming problem formalized by the computation of the Wasserstein distance (a.k.a. Earth Mover's distance (EMD)) which is the Kantorovich-Rubinstein (KR) transshipment problem.

$$\dot{\mu}_c = \inf_q \int ||x-y||^s q(x,y)dxdy$$

This is known to have a computational complexity of $\mathcal{O}(N^3 \log N)$ where N is the number of nodes[SJ08]. The authors in [SJ08] presented that the dual of the KR problem

$$\dot{\mu}_c = \sup_f \int f(x)(p_1(x)-p_2(x))dx$$

with the same optimal value has a wavelet domain representation

$$d_{wemd}(p) = \hat{\mu}_c = C_0 \sum_k |p_k| + C_1 \sum_\lambda 2^{-j(s+n/2)}|p_\lambda|$$

(with constants set to $C_0 = 0$ and $C_1 = 1$) and showed that this wavelet EMD metric is equivalent to EMD. The same analysis applies for discrete distributions. The Wasserstein distance computational time calculation can be improved to linear time $\mathcal{O}(N)$ complexity via the wavelet EMD approximation method while preserving the performance of the EMD.

2.3.2 Ollivier-Ricci curvature bounds

In addition, [JL14] showed a general sharp inequality for the ORC bounds for a locally finite undirected, weighted and connected graph, as follows

$$\begin{aligned}
\kappa(ij) \geq & - \left(1 - \frac{\rho_{ij}}{d_i} - \frac{\rho_{ij}}{d_j} - \sum_{i_1, i_1 \sim i, i_1 \sim j} \frac{\rho_{i_1,i}}{d_i} \vee \frac{\rho_{i_1,j}}{d_j}\right)_+ \\
& - \left(1 - \frac{\rho_{ij}}{d_i} - \frac{\rho_{ij}}{d_j} - \sum_{i_1, i_1 \sim i, i_1 \sim j} \frac{\rho_{i_1,i}}{d_i} \wedge \frac{\rho_{i_1,j}}{d_j}\right)_+ \\
& + \sum_{i_1, i_1 \sim i, i_1 \sim j} \frac{\rho_{i_1,i}}{d_i} \wedge \frac{\rho_{i_1,j}}{d_i} \\
\kappa(x,y) \leq & \sum_{i_1, i_1 \sim i, i_1 \sim j} \frac{\rho_{i_1,i}}{d_i} \wedge \frac{\rho_{i_1,j}}{d_j}
\end{aligned}$$

where ρ_{ij} is the edge weight between node i and j; $a_+ = \max(a,0)$; $a \wedge b = \min(a,b)$; $a \vee b = \max(a,b)$; $d_i = \sum_{j \sim i} \rho_{ij}$; $j \sim i$ denotes the existence of the edge between i and j. The results in [JL14, BJL11] illustrate the relation between the Ollivier-Ricci curvature, the clustering coefficient, and the graph Laplacian.

Algorithm	Complexity	Average accuracy*	References
Ollivier-Ricci Curvature	$\mathcal{O}(EV^2)^\dagger$	0.990	
Edge Betweenness	$\mathcal{O}(E^2N)$	0.830	[GN02]
Fast Greedy	$\mathcal{O}(N\log^2(N))$	0.075	[CNM04]
Label Propagation	$\mathcal{O}(E)$	0.645	[RK]
Leading Eigenvector	$\mathcal{O}(N(E+N))$	0.930	[New06]
Spinglass	$\mathcal{O}(N^{3.2})$	0.910	[RB06]
Walktrap	$\mathcal{O}(EN^2)$	0.990	[PL05]

Table 1: Computational time complexity summary for some commonly used community detection algorithms, where N is the number of nodes, E is the number of edges, and V is the average degree with $V \ll E$. †The ORC-based CI time complexity based on the wavelet EMD approximation approach (see Sec. 2.3.1). *Based on SBM generated networks with parameters $(k, l, p_{in}, p_{out}) = (15, 10, 0.7, 0.5)$ with prediction accuracy defined as the percentage of the number of correctly identified communities over all ground truth communities.

2.3.3 Time complexity table

Table 1 shows the summary of the computational time complexities and prediction accuracies of some commonly used community detection algorithms. Average prediction accuracy run on a benchmark SBM generated network with $(k, l, p_{in}, p_{out}) = (15, 10, 0.7, 0.5)$ with prediction accuracy defined as the percentage of the number of correctly identified communities over all ground truth communities. Apart from the proposed ORC-based CI, all the community detection algorithms are based on the `igraph`[CN14] python implementation.

3 Additional Results and Discussion

3.1 Stochastic Block Model (SBM)

For artificial networks, we check the performance according to the following SBM generator parameters: size per community (k), number of communities (l), and intra- and inter-community probabilities (p_{in}, p_{out}).

Each algorithm's performance accuracy is evaluated by comparing the ground truth set of communities versus the identified set of communities. When a set of labelled nodes matches the members of one of the ground truth community sets, this is considered a correctly identified community. The prediction accuracy is obtained as a percentage of the correctly identified communities over all ground truth communities.

3.1.1 Intra- and inter-cluster densities vs. prediction accuracy

Figure 2 shows the prediction accuracy of the ORC-based, LEM and EM CIs on SBMs based on varying only one of the SBM generating parameters: size per community (a), number of communities(b), intra-community probability (c), and inter-community probability (d) (while keeping all other parameters fixed).

As we vary the size per community while keeping the number of communities and the probability of intra- and inter-community edges between any two nodes (p_{in} and p_{out}) constant (see Fig. 2a), we observe the accuracy of the ORC-based CI improves significantly for networks with more than 7 communities. The low accuracy for sizes k less than 7 is due to the high probability to merge two small-sized communities caused by the addition of inter-community edges between the two communities. For sizes of community less that 15, the ORC-based CI performs better compared to both LEM and EB methods. There is also a slight degradation in accuracy for the LEM- and EB-based CIs after community size greater than 30.

As we vary the number of communities but keeping the size per community and the probability of intra- and inter-community edges between any two nodes (p_{in} and p_{out}) constant (see Fig. 2b), we observe the accuracy of the ORC-based CI degrades significantly for networks with more than 35 communities. In contrast, the LEM demonstrates worse performance as its accuracy degrades significantly for more than 15 communities. Both, the ORC- and LEM-based CI reach

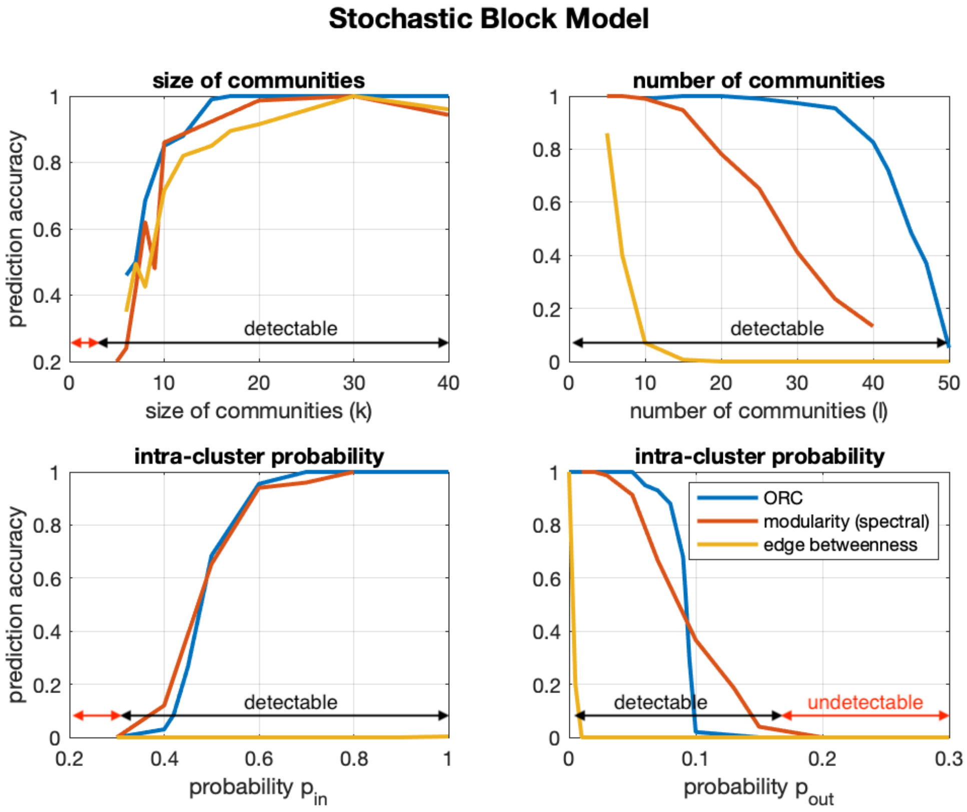

Figure 2: Prediction accuracy of the ORC-based, LEM and EB CIs on SBMs based on varying size per community and number of communities parameters. **(a)** Prediction accuracy as a function of size of communities for $l = 10$ number of communities. **(b)** Prediction accuracy as a function of number of communities for $k = 20$ size per community. **(c)** Prediction accuracy as a function of intra-cluster probability p_{in} for fixed $p_{out} = 0.05$. **(d)** Prediction accuracy as a function of inter-cluster probability p_{out} for fixed $p_{in} = 0.7$. Intra- and inter-community edge wiring probabilities are set to $p_{in} = 0.7$ and $p_{out} = 0.05$ for subplots (a) and (b). Size of community and number of communities are set to $k = 20$ and $l = 30$ for subplots (c) and (d). In addition, the theoretical SBM detectability regimes are indicated in all four subplots with its respective detectability thresholds: (a) $k^* \geq 3$, (b) $l^* \leq 156$, (c) $p^*_{in} \geq 0.35$, and (d) $p^*_{out} \leq 0.17$. The black horizontal double arrow indicates the detectable regime, while the red horizontal double arrow indicates the undetectable regime.

50% accuracy for more than 30 communities. The EB-based CI performs the worst out of the three CI algorithms.

Meanwhile, Figs. 2c and 2d show the prediction accuracy as we vary the intra- and inter-community probabilities for fixed number of communities $l = 30$ and size per community $k = 20$. In Fig. 2c, both the ORC-based and LEM-based CI almost have the same performance for varying p_{in} values. In Fig. 2d, the LEM-based CI performs worse compared to the ORC-based CI for $p_{out} < 0.1$. Better performance for for $p_{out} > 0.1$. Simulation results show that the ORC-based CI provides better or comparable accuracy with the LEM-based CI.

Additionally, the theoretical SBM detectability regimes are indicated in all four subplots of Fig. 2 with its respective detectability thresholds: (a) $k^* \geq 3$, (b) $l^* \leq 156$, (c) $p^*_{in} \geq 0.35$, and (d) $p^*_{out} \leq 0.17$. The black horizontal double arrow indicates the detectable regime, while the red horizontal double arrow indicates the undetectable regime. The analysis of the prediction accuracy performance with respect to the SBM detectability regime is discussed in the next section.

3.1.2 Detectability regime in stochastic block models

In [DKMZ11], the paper explored the SBM detectability landscape for the ratio of inter- and intra-cluster densities. The results in Fig. 2 are shown in terms of the SBM detectable regime satisfying $|c_{in} - c_{out}| > q\sqrt{c}$, where c_{in}, c_{out}, q, c are the intra- and inter-cluster affinities, number of communities (of the same size), and average degree, respectively. For $|c_{in} - c_{out}| < q\sqrt{c}$, the generated graph from the stochastic block model is indistinguishable from a random graph. In our notation, $c_{in} = Np_{in}$, $c_{out} = Np_{out}$, $N = lk$ or the total number of nodes, $q = l$ or the number of communities, and the average degree c is approximated to $c = kp_{in} + (N - k)p_{out}$, with k as the size per community. Fig. 3 illustrates the same results shown in Fig. 2(c) but in terms of the density ratio p_{out}/p_{in}. In addition, both the detectable and undetectable regimes are highlighted according to the conditions in [DKMZ11]. For varying p_{in}, the detectable regime lies in $\epsilon^* = (p_{out}/p_{in})^* < 0.1429$ or for $p^*_{in} > 0.35$. Similarly, Fig. 4 illustrates the same results shown in Fig. 2(d) in terms of the density ratio p_{out}/p_{in} with the detectable and undetectable regimes. For varying p_{out}, the detectable regime lies in $\epsilon^* = (p_{out}/p_{in})^* < 0.2429$ or for $p^*_{out} < 0.17$.

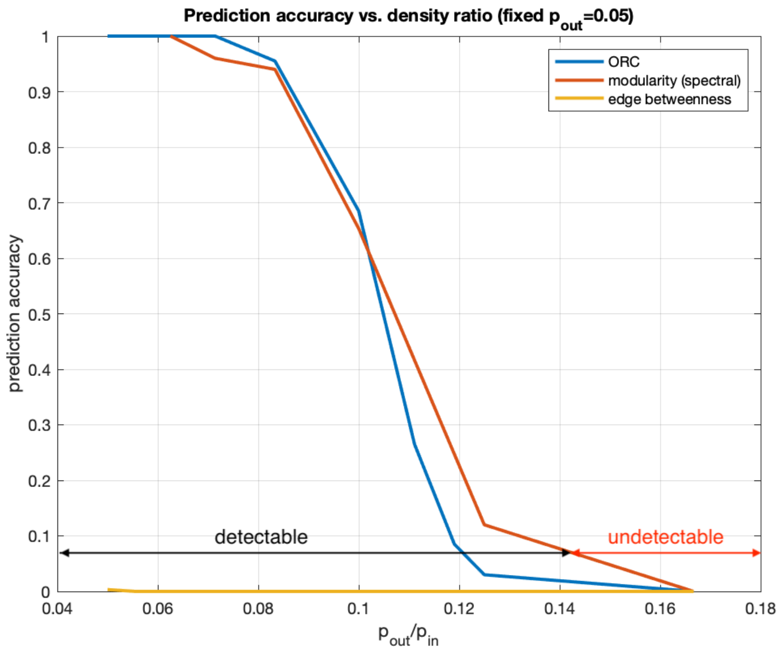

Figure 3: Prediction accuracy of the ORC-based, LEM and EB CIs on SBMs based vs. the density ratio p_{out}/p_{in} for fixed $p_{out} = 0.05$. The detectability threshold $\epsilon^* = 0.1429$ or $p^*_{in} = 0.35$.

Supplementary References

[BHJ09] M Bastian, S Heymann, and M Jacomy. Gephi: An open source software for exploring and manipulating networks. *International AAAI Conference on Weblogs and Social Media*, pages 361–362, 2009.

[BJK12] Reza Banirazi, Edmond Jonckheere, and Bhaskar Krishnamachari. Heat diffusion algorithm for resource allocation and routing in multihop wireless networks. *GLOBECOM - IEEE Global Telecommunications Conference*, pages 5693–5698, 2012.

[BJK14a] R Banirazi, E Jonckheere, and B Krishnamachari. Dirichlet's principle on multiclass multihop wireless networks: Minimum cost routing subject to stability. *MSWiM 2014 - Proceedings of the 17th ACM International Conference on Modeling, Analysis and Simulation of Wireless and Mobile Systems*, pages 31–40, 2014.

[BJK14b] Reza Banirazi, Edmond Jonckheere, and Bhaskar Krishnamachari. Heat-Diffusion: Pareto optimal dynamic routing for time-varying wireless networks. *Proceedings - IEEE INFOCOM*, pages 325–333, 2014.

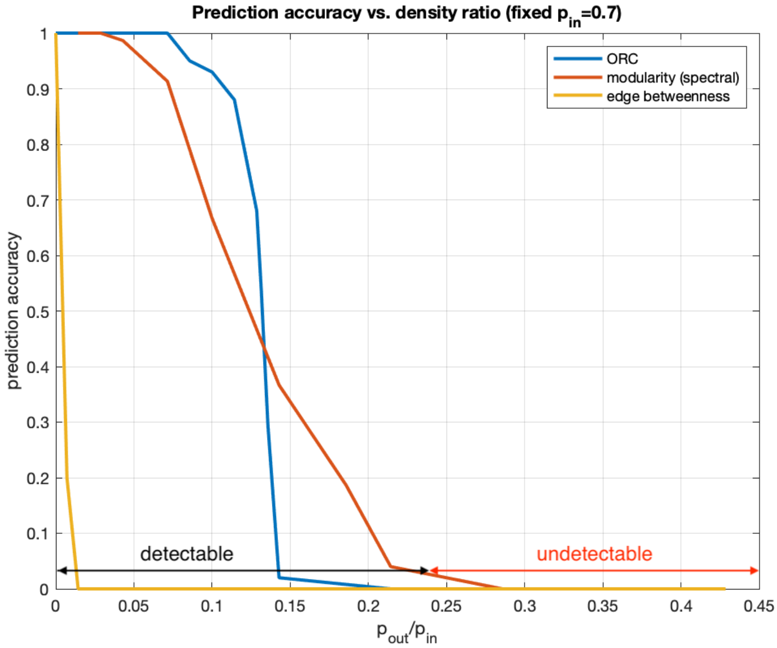

Figure 4: Prediction accuracy of the ORC-based, LEM and EB CIs on SBMs based vs. the density ratio p_{out}/p_{in} for fixed $p_{in} = 0.7$. The detectability threshold $\epsilon^* = 0.2429$ or $p^*_{out} = 0.17$.

[BJK14c] Reza Banirazi, Edmond Jonckheere, and Bhaskar Krishnamachari. Minimum delay in class of throughput-optimal control policies on wireless networks. *Proceedings of the American Control Conference*, pages 2668–2675, 2014.

[BJL11] Frank Bauer, Jürgen Jost, and Shiping Liu. Ollivier-Ricci curvature and the spectrum of the normalized graph Laplace operator. pages 1–20, 2011.

[CN14] Gábor Csárdi and Tamás Nepusz. The igraph software package for complex network research. *Journal of Computer Applications*, (November 2005):9, 2014.

[CNM04] Aaron Clauset, M. E.J. Newman, and Cristopher Moore. Finding community structure in very large networks. *Physical Review E - Statistical Physics, Plasmas, Fluids, and Related Interdisciplinary Topics*, 70(6):6, 2004.

[DKMZ11] Aurelien Decelle, Florent Krzakala, Cristopher Moore, and Lenka Zdeborová. Asymptotic analysis of the stochastic block model for modular networks and its algorithmic applications. *Physical Review E - Statistical, Nonlinear, and Soft Matter Physics*, 84(6):1–19, 2011.

[GN02] M. Girvan and M. E. J. Newman. Community structure in social and biological networks. *Proceedings of the National Academy of Sciences*, 99(12):7821–7826, 2002.

[HSS08] Aric A Hagberg, Daniel A. Schult, and Pieter J. Swart. Exploring network ntructure, nynamics, and nunction using NetworkX. *Proceedings of the 7th Python in Science Conference*, (SciPy):11–15, 2008.

[JL14] Jürgen Jost and Shiping Liu. Ollivier's Ricci Curvature, Local Clustering and Curvature-Dimension Inequalities on Graphs. *Discrete and Computational Geometry*, 51(2):300–322, 2014.

[JLBB11] Edmond Jonckheere, Mingji Lou, Francis Bonahon, and Yuliy Baryshnikov. Euclidean versus hyperbolic congestion in idealized versus experimental networks. *Internet Mathematics*, 7(1):1–27, 2011.

[LRO+17] Wuchen Li, Ernest K Ryu, Stanley Osher, Wotao Yin, and Wilfrid Gangbo. A Parallel Method for Earth Mover's Distance. *Journal of Scientific Computing*, 2017.

[New06] M. E.J. Newman. Finding community structure in networks using the eigenvectors of matrices. *Physical Review E - Statistical, Nonlinear, and Soft Matter Physics*, 74(3), 2006.

[NLG+15] Chien-Chun Ni, Yu-Yao Lin, Jie Gao, Xianfeng David Gu, and Emil Saucan. Ricci Curvature of the Internet Topology. *2015 IEEE Conference on Computer Communications (INFOCOM)*, 26:2758–2766, jan 2015.

[Oll09] Yann Ollivier. Ricci curvature of Markov chains on metric spaces. *J. Functional Analysis*, 256(3):810–864, 2009.

[PL05] Pascal Pons and Matthieu Latapy. Computing communities in large networks using random walks. *Lecture Notes in Computer Science (including subseries Lecture Notes in Artificial Intelligence and Lecture Notes in Bioinformatics)*, 3733 LNCS:284–293, 2005.

[RB06] Jörg Reichardt and Stefan Bornholdt. Statistical mechanics of community detection. *Physical Review E - Statistical, Nonlinear, and Soft Matter Physics*, 74(1):1–16, 2006.

[RK] Usha Nandini Raghavan and Soundar Kumara. Near linear time algorithm to detect community structures in large-scale networks 1 1. pages 1–12.

[SJ08] Sameer Shirdhonkar and David W. Jacobs. Approximate earth mover's distance in linear time. *26th IEEE Conference on Computer Vision and Pattern Recognition, CVPR*, 2008.

[WJB14] Chi Wang, Edmond Jonckheere, and Reza Banirazi. Wireless network capacity versus Ollivier-Ricci curvature under Heat-Diffusion (HD) protocol. *Proceedings of the American Control Conference*, pages 3536–3541, 2014.

[WJB16] Chi Wang, Edmond Jonckheere, and Reza Banirazi. Interference constrained network control based on curvature. *Proceedings of the American Control Conference*, 2016-July:6036–6041, 2016.
